# Reassessing the non-linear causal link between long-duration commuting and self-rated health: do behavioral preferences and built environment matter?

**DOI:** 10.3389/fpubh.2024.1452014

**Published:** 2024-10-02

**Authors:** Ning Qiu, Wen Li, Danrui Cui, Mengbing Du, Zibo Xing, Dongxu Cui, Han Xinyu

**Affiliations:** ^1^School of Architecture and Urban Planning, Shandong Jianzhu University, Jinan, China; ^2^Yellow River Institution, Shandong Jianzhu University, Jinan, China; ^3^Architecture College of Xi’an, Xi’an University of Architecture and Technology, Xi’an, China; ^4^School of Political Science & Public Administration, Wuhan University, Wuhan, China; ^5^Beijing Municipal Institute of City Planning & Design, Beijing, China

**Keywords:** commuting time, self-rated health, group heterogeneity, active commuting, physical activity, perceived built environment

## Abstract

**Introduction:**

Long-duration commuting is widely recognized for its significant influence on health. However, while research has traditionally focused on direct impacts, there remains a critical need to explore the nonlinear dynamics of this relationship. This study aims to deepen our understanding of how behavioral preferences and built environments contribute to these complex interactions.

**Methods:**

This study was conducted in Jinan, China’s most congested city, using data from the “Jinan Residents Commuting Survey” of 1,755 participants aged 19 to 59. We applied Generalized Propensity Score Matching (GPSM) to explore the nonlinear effects of commuting time on self-rated health, adjusting for participants’ sociodemographic characteristics. Variables related to active commuting, physical activity, and perceived built environment were also examined for their potential moderating effects.

**Results:**

Commuting for less than 21 minutes enhances health, but negative effects intensify and peak at 60 minutes. Heterogeneity analysis reveals that women and older adults, especially those with higher incomes, are more susceptible to long commutes, experiencing a delayed onset of adverse effects. While active commuting offers health benefits, it may exacerbate health issues if prolonged. Conversely, regular physical activity consistently improves health outcomes related to commuting. Additionally, factors like residential greenery and walkability help alleviate commuting-related stress, improving the overall commuting-health dynamic.

**Discussion:**

This study clarifies the commuting-health relationship by identifying key time thresholds and the positive effects of active commuting and physical activity on mitigating longer commute impacts. The findings inform healthier commuting behaviors and offer practical guidelines for urban planning and policy-making to enhance commuter well-being.

## 1 Introduction

In the context of rapid urbanization, long-duration commuting has become a daily reality for many urban residents (1). While commuting is necessary to connect residential settings with workplaces, studies indicate that prolonged commuting can impact individual health adversely (2). As commuting time extends, residents may face increased psychological stress (3), physical fatigue and health risks associated with sedentary behaviors (4), collectively affecting cardiovascular health (1), mental well-being (5), and overall quality of life (6). Consequently, numerous urban transportation and residential policies are being developed around the commuting-health nexus, including the optimization of public transit systems (7), promotion of green transportation options (8), and the advancement of health-oriented urban design (8).

These impacts demonstrate significant heterogeneity across different population groups (8). For instance, lower-income individuals, the older adult, and women may be more susceptible to the adverse effects of long-duration commuting (9). Moreover, individual behavioral preferences, such as active commuting modes (e.g., walking and cycling) (10) and regular physical exercise (10), are recognized as potential mitigating factors against some of the negative health effects associated with commuting. Additionally, factors related to the built environment, including greenery (11), walkability (12), and traffic safety (13) are acknowledged as crucial determinants. These elements not only encourage healthier commuting practices but also directly enhance the quality of life and physical health of residents.

However, current research on the impact of commuting on health often exhibits three significant gaps. Firstly, numerous studies have confirmed the nonlinear effects of commuting distance or duration on health (14, 15), but these studies typically do not establish causality. Consequently, they may be subject to issues of endogeneity and limitations inherent to their statistical methodologies, which impede accurate estimation of causal effects. Secondly, the impact on different demographic groups may vary, including potential differences in inflection points and trends in the effects, which may differ significantly among populations, complicating the formulation of differentiated transportation and residential policies. Thirdly, while the health benefits of healthier behavioral preferences and built environments are known (16, 17), further research is needed to determine how these factors moderate the commuting-health relationship. This is essential for creating specific interventions to foster healthier commuting practices and reduce health risks.

To address these gaps, this study selects Jinan, a typical city characterized by long-distance commuting, to investigate the causal relationships between commuting and health using the Generalized Propensity Score Matching (GPSM) method. Specifically, this research aims to resolve three key research questions: Firstly, it explores the existence and dynamics of a causal relationship between long-duration commuting and health. Secondly, it investigates how commuting impacts health across various demographic groups, including different incomes, ages, and genders. Thirdly, the study examines the moderating roles of residents’ behavioral preferences and built environments in the commuting-health nexus. Through this analysis, we aim to provide insights that will aid urban planners and policymakers in designing healthier and more sustainable urban transportation systems.

## 2 Literature review

### 2.1 Non-linear commuting-health nexus: the rising debate in time thresholds

Extensive studies consistently link long-duration commuting with adverse health effects, particularly emphasizing the differential health impacts associated with commuting time thresholds. On one hand, prolonged commuting is directly correlated with psychological health issues. For instance (1), indicates that individuals experience a significant increase in psychological stress when commuting times exceeds 45 min, identifying this duration as a critical threshold for psychological health deterioration. Similarly (18), found that motorists who commute longer than 60 min had a higher risk of mental distress. On the other hand, long-duration commuting is also directly linked to physical health problems such as cardiovascular diseases and obesity (19). Found that commuting for more than 60 min leads to decreased life satisfaction, while commuting for over 90 min significantly increases the risk of obesity. Moreover, commuters who are exposed to pollutants during their travels are at increased risks of chronic respiratory diseases and cardiovascular conditions, especially those who commute for extended periods (20). Conversely, shorter commuting times have been shown to positively impact self-rated health. On the other hand (62) demonstrated that even slight increases in weekly commuting distances, such as a few additional kilometers, could significantly improve self-rated health.

Overall, these findings highlight the public health significance of commuting time thresholds. Commuting durations exceeding 45 min begin to negatively impact psychological health, while durations over 90 min have substantial adverse effects on physical health. However, it is important to note that both of these time points might be relatively long for cities of average size, where the longest average commuting time is usually around 40 min, and times exceeding 60 min may be defined as extreme commuting (21). Generally, the “happiness commute “time is considered to be around 20 min, which is likely to have a positive impact on health (63). While these studies reveal correlations between commuting time and health and identify specific thresholds, they fail to demonstrate the variability in impact as commuting durations continue to change. Moreover, the conclusions of these studies largely rely on correlational data rather than causation. Future research should employ more precise methodologies to probe the continuous variations and better understand how specific commuting times influence health. As a result, merely examining the correlation between commuting time and health does not capture the continuous variation in health relative to commuting duration or the variability in this relationship’s slope. Furthermore, definitions of poor health vary widely, encompassing conditions from stress and unhappiness to severe physical illnesses like cardiovascular diseases. In conclusion, to truly grasp the complexities of how commuting affects health, it is essential to go beyond correlations and explore the detailed changes that occur over varying commute lengths.

### 2.2 Population heterogeneity: inequality issues in commuting stress

The impact of long-duration commuting on health exhibits significant variability across different demographic characteristics, particularly age (22), gender (3), and income levels (23), resulting in notable disparities in health outcomes. For example, young individuals may better adapt to the stress associated with long commutes (24), but for middle-aged and older adults, prolonged commuting can exacerbate cardiovascular diseases and other health issues.

Gender differences also play a crucial role in the health impacts of commuting. Female commuters are more vulnerable to the negative effects of long commutes, such as increased psychological stress and overall health decline (5). This could be attributed in part to distinct social and familial roles, as women often bear greater domestic responsibilities, with long commutes intensifying their stress burden (25). Conversely, some studies highlight the health risks faced by men in long commutes, such as a heightened risk of cardiovascular diseases, which relate to higher work stress and lifestyle factors during commuting, like unhealthy eating habits and lower levels of physical activity.

Regarding income levels, individuals with lower incomes often have limited options for choosing healthier and more comfortable commuting methods (26). Prolonged commuting may restrict access to healthy food and engagement in physical activities for this demographic, thereby increasing the risk of health issues (27). Studies have shown that when commuting time exceeds 45 min, the psychological stress and physical health problems of low-income residents significantly increase. These findings suggest that policymakers need to consider the combined influences of gender, age, income, and commuting duration, and how these factors interact across different populations to effectively mitigate the negative health impacts of long-duration commuting.

### 2.3 Moderating roles of behavioral preferences: commuting modes and physical activity

Extensive research has demonstrated that active commuting and regular physical activity positively impact health (28), enhancing cardiovascular health (29), reducing stress levels, and improving overall quality of life (30). Studies consistently show that individuals who walk or cycle to work have a lower risk of cardiovascular diseases, obesity, and diabetes compared to those who commute by car or public transportation (31). For instance, a meta-analysis found that active commuters had an 11% reduction in cardiovascular risk (32). The physical activity involved in active commuting helps achieve daily exercise goals, promotes cardiovascular health, strengthens muscles, and boosts metabolic functions (33). Moreover, regular physical activity, whether incorporated into daily routines like commuting or as part of structured exercise programs, significantly reduces the risk of chronic diseases. The World Health Organization recommends at least 150 min of moderate-intensity aerobic activity weekly for health benefits and disease prevention (4).

The synergistic effect between active commuting and regular physical activity also extends to mental health (34). Regular participation in physical activity is known to reduce symptoms of depression and anxiety, improve mood, and enhance self-esteem. Active commuting contributes to these benefits by providing regular, unplanned physical activities that can reduce stress and increase overall life satisfaction (35). Guell et al.’s (36) research emphasized that the routine nature of active commuting can help establish a daily routine that fosters a positive psychological state.

However, the moderating role of these activities in mitigating the negative health impacts of long-duration commuting has not yet been clearly established. Some views suggest that while active commuting and physical activities are beneficial for health, their benefits may be diminished under extreme commuting conditions, such as very long commuting distances. This perspective requires further empirical research, particularly through long-term tracking and large-scale studies, to explore the true impact of active commuting and physical activity across different commuting distances.

### 2.4 Moderating roles of built environments: from the perspective of individual perception

Numerous studies have demonstrated that various characteristics of the built environment, such as greenery (11), accessibility (12), and pedestrian friendliness (13), significantly influence individuals’ commuting choices and their associated health outcomes. The impact of the built environment on the commuting-health relationship primarily manifests through its potential to guide individual behaviors (37). For instance, a well-designed pedestrian environment and bicycle facilities can encourage residents to choose walking or cycling over driving, which not only reduces reliance on automobiles but also increases daily physical activity (38), thereby improving cardiovascular health and reducing obesity risks (39). One important factor is green space, such as parks and green streets, are pivotal for health, providing essential contact with nature that alleviates psychological stress, enhances mood, and promotes physical health. Residents in high-greenery areas report lower stress and better psychological well-being and are more likely to engage in outdoor activities such as walking and cycling, boosting physical activity (40). Another critical factor is transit accessibility, which can lessen commuting stress and reduce mental health issues associated with long-duration commuting (2). Improved public transit access specifically eases the negative mental health impacts of prolonged travel times.

Despite the insights provided by existing research, there are still notable gaps. Firstly, most studies do not differentiate between the built environment features of workplace and residential settings, though each can differently influence commuting behavior and health outcomes. For example, the green environment at a residential location may more directly affect residents’ leisure activities and stress levels (41), while the transportation design at the workplace might crucially determine the choice of commuting mode. Moreover, current research often overlooks the discrepancies between residents’ perceptions of their environment and the actual environmental features. Discrepancies between perceived and actual greenery (42), the density of pedestrian pathways versus the walking experience (43), and the perceived versus actual transit accessibility (44), road density, and station proximity can all impact the accuracy of research findings and the effectiveness of related policies. For instance, even if the greenery rate is high, if residents do not perceive it, the positive psychological and behavioral effects may not materialize (45). Given these research gaps, future studies need to more carefully distinguish between the built environment characteristics of workplace and residential settings and consider how these independently and jointly affect commuting behavior and health outcomes. Study should also focus more on residents’ subjective perceptions of the built environment, exploring how these perceptions align with objective environmental parameters and their comprehensive impact on health.

**Figure 1:**
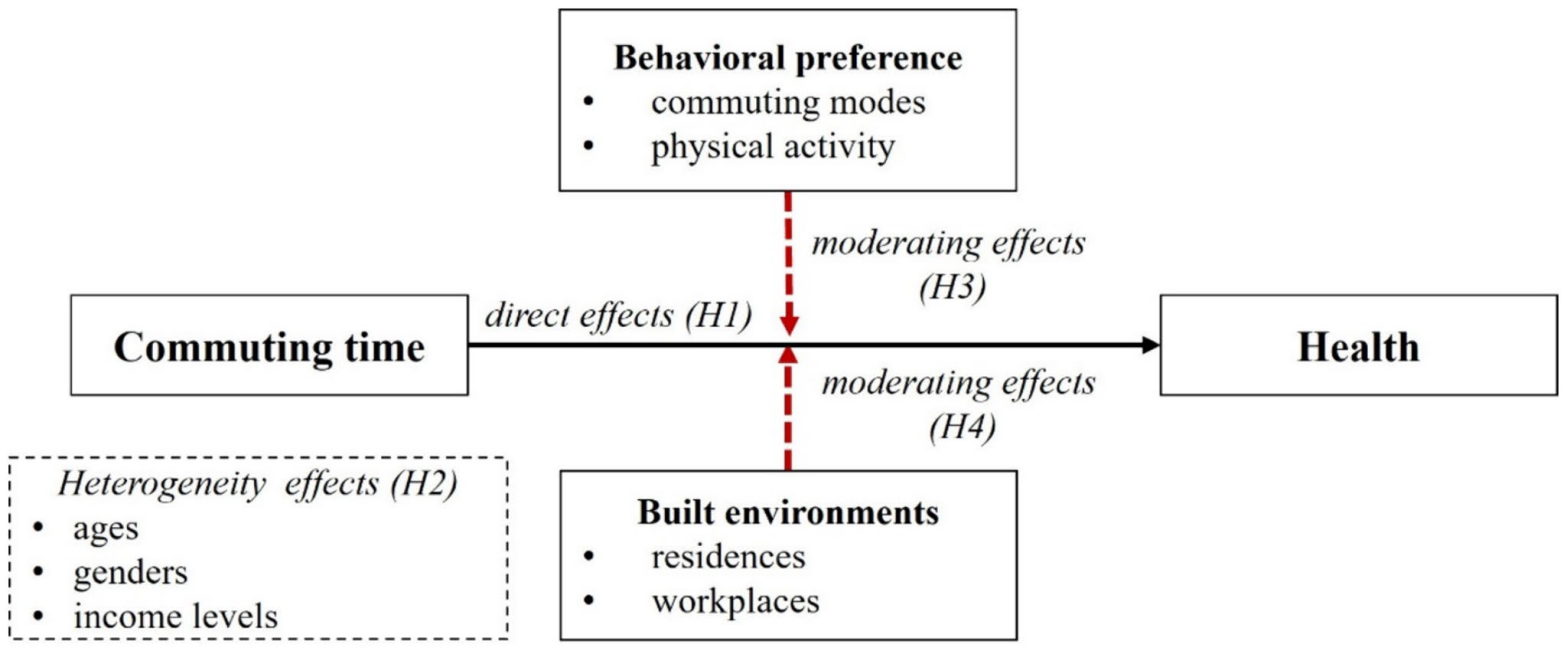
Theoretical framework and research hypothesis.

Accordingly, Theoretical framework and hypothesis are proposed as follows (Figure 1).

*H1*: Long-duration commuting adversely affects residents’ health. When residents exceed a certain commuting duration threshold, their health conditions may deteriorate.

*H2*: The impact of long-duration commuting on health varies across different ages, genders, and income levels. Residents from middle and lower socio-economic backgrounds, older residents, or females may be more vulnerable to the negative health effects of long-duration commuting.

*H3*: The impact of long-duration commuting on residents’ health is partially moderated by their mode of commuting and physical activity levels. Active commuting and regular physical activity engagement may mitigate the adverse health effects associated with long-duration commuting.

*H4*: The impact of long-duration commuting on residents’ health is partially moderated by the built environment of their residential and workplace areas. Features such as enhanced greenery, walkability, safety, and traffic environment can mitigate the adverse health effects of long-duration commuting.

## 3 Data and methods

### 3.1 Data and variables

In the empirical analysis, we focus on the urban area of Jinan, a city known for its congestion and long-duration commutes. According to the “2022 Annual Report on Commuting in Major Chinese Cities,” only 54% of Jinan’s commuters experience “happiness commute” within a 5-kilometer radius, while more than 10% endure commutes exceeding 60 min. The “Jinan Residents Commuting Survey,” conducted in December 2023, gathered data from 1,755 participants, aged 19 to 59, using mobile devices. This survey captured detailed information on socio-economic characteristics, job-housing locations, commuting behaviors, physical activities, and self-rated health.

The variables descriptions and definitions are shown in Table 1. The dependent variable, *Self-rated Health*, is assessed based on prior research (46). This study measures Self-rated Health using a Likert scale, with responses ranging from “very bad” (1) to “very good” (5). To ensure clarity and consistency, participants were queried about the frequency of experiencing mental health issues such as anxiety or physical health problems like chronic pain over the past year. Responses were scored on a scale from “never” (1) to “always” (5). The primary independent variable in this study is *commuting time*, which varies from 10 to 90 min.

**Table 1:** Summary statistics.

Variables		Definition	Mean	Std dev
Self-rated health		Self-rated health status of the respondents? 5-point scale: from 1 = very bad to 5 = very good	3.27	1.15
Commuting time		One-way commute time (minutes)	33.85	17.89
Commuting distance		One-way commute length (km)	8.78	9.61
Male		0 = female, 1 = male	0.44	0.50
Age		Age	37.45	11.76
Marital status dummies		1 = unmarried, 2 = married, 3 = divorced	–	–
Education level		Educational attainment: 1 = Junior high school and below, 2 = Senior high school and below, 3 = undergraduate college, 4 = graduate student and above	3.17	0.51
Non-agricultural household registration		0 = agricultural household registration, 1 = non-agricultural household registration	1.36	0.49
Occupation type dummies		1 = Public institutions, 2 = workers,3 = white-collar workers 4 = enterprises,5 = service industries 6 = individual businesses,7 = full-time,8 = freelance	–	–
Car ownership		Whether has car	0.77	0.42
Income		Individual income (thousand yuan)	2.41	1.59
Active commuting		Active commuting levels: 1 = car, 2 = public transit,3 = electric bike,4 = walk, bicycle	2.05	1.03
Physical activity		Weekly fitness frequency (times) * Duration of each workout (minutes)	3.68	4.23
Perceived built environment satisfaction (5-point scale: from 1 = very dissatisfied to 5 = very satisfied)	Workplace greenery	How satisfied are respondents with greenery near their workplace?	3.78	1.08
	Workplace walkability	How satisfied are respondents with the walking environment near their workplace?	3.67	1.17
	Workplace Safety	How satisfied are respondents with safety near their workplaces?	4.11	0.94
	Workplace Accessibility	How satisfied are respondents with the ease of transportation near their workplace?	3.60	1.29
	Residential Greenery	How satisfied are respondents with greenery near where they live?	3.69	1.15
	Residential Walkability	How satisfied are respondents with the walking environment near where they live?	3.68	1.14
	Residential Safety	How satisfied are respondents with the safety near where they live?	3.85	1.10
	Residential Accessibility	How satisfied are respondents with the ease of transportation near where they live?	3.61	1.24

This study controlled for a number of variables, including gender, generation, marital status, education level, household registration, occupation type and income, to comprehend the sociodemographic characteristics of participants. Previous research has consistently shown that individuals with agricultural household registration, low income, and lower educational levels are more likely to experience long-duration commuting.

Two sets of variables define the behavioral preferences of participants in this study: active commuting and physical activity. The former variable was grouped into Passive commuting (car), Public transport (bus and metro), E-bike and Active commuting (Walking and Bike). To reflect their level of physical activity, these categories were assigned scores from 1 to 4, with higher scores indicating more active commuting (47). The scoring system aims to represent the relative activity levels rather than precise multiplier relationships. For instance, walking and cycling generally involve more physical activity than driving or using public transit. The estimates are based on average activity durations: walking/cycling (150 min/week), e-bike (100 min/week), public transit (50–100 min/week), and car (25–50 min/week), according to the latest WHO physical activity standards, which suggest increasing activity levels per week to achieve significant health benefits. For this study, exercise is defined as a subset of physical activity that is planned, structured, and repetitive, aimed at improving or maintaining. Participants were asked, “How often do you engage in exercise, such as recreational leisure fitness, per week?” and “How long do you spend on each fitness?” Physical activity intensity is the product of activity frequency and the duration of a single activity.

The regression analysis incorporated eight perceived built environment variables to control for external environmental conditions, specifically examining greenery, walkability, safety, and accessibility in both residential and workplace settings. Participants rated their satisfaction with these elements on a scale from 1 (very dissatisfied) to 5 (very satisfied), reflecting their perceptions of the built environment.

### 3.2 Model specifications

(1) OLS regression model

In order to estimate the impact of commuting time on self-rated health and judge the nonlinear relationship between the two, the following linear model is designed in this study, namely Equation 1:


(1) $$SRH_{i}=\alpha_{0}+\alpha_{1}\;\mathrm{commutingtim}e_{i}+\alpha_{2}\mathrm{contro}l_{i}+\varepsilon_{i}$$

In the formula, explained variable SRH refers to self-rated health; commuting time is the core explanatory variable of this study. Coefficient $\alpha_{1}$ refers to commuting time coefficient, indicating the commuting time’s effect on self-rated health. $\mathrm{contro}l_{i}$ is the control variable, and other factors that may affect self-rated health are included in this study, such as age, gender and education level. $\varepsilon_{i}$ is a random error term.

(2) Generalized propensity score matching

The analysis of treatment effects primarily relies on the “counterfactual” framework by (48). However, propensity score matching (PSM) is limited to binary treatment variables. For example, treating commuting time as either less than 1 h (0) or more than 1 h (1) as two distinct categories simplify the variable’s nuanced impact on health too much (49). Expanded this framework to accommodate multivariate or continuous treatment variables, introducing Generalized Propensity Score (GPS) matching. This method better captures the varying outcomes at different treatment intensities, addressing PSM’s limitations.

GPSM was further evaluated in three steps. First, estimate the conditional probability density of treatment intensity based on the covariates *X*, and employ the Fractional Logit model to adjust the density function for estimation, namely Equation 2.


(2) $$\begin{array}{l} E\left(T_{i}|X_{i}\right)=F\left(X_{i}\beta \right)=\frac{\exp\left(X_{i}\beta \right)}{1+\exp\left(X_{i}\beta \right)};\\ \hat{R_{i}}={\left[F\left(X_{i}\hat{\beta }\right)\right]}^{T_{i}}\cdot {\left[1-F\left(X_{i}\hat{\beta }\right)\right]}^{1-T_{i}} \end{array}$$

Then, the conditional expectation model of output variables (this study mainly refers to individual self-rated health) is constructed by using processing variables and generalized propensity score variables, namely Equation 3:


(3) $$\begin{array}{l} E\left(Y_{i}|T_{i},{\hat{R}}_{i}\right)=\alpha_{0}+\alpha_{1}T_{i}+\alpha_{2}{T_{i}}^{2}+\alpha_{3}{T_{i}}^{3}+\alpha_{4}{\hat{R}}_{i}\\ \;+\alpha_{5}{{\hat{R}}_{i}}^{2}+\alpha_{6}{{\hat{R}}_{i}}^{3}+\alpha_{7}T_{i}{\hat{R}}_{i} \end{array}$$

Thirdly, Estimate the “Average Dose–response Function” $\mu \left(t\right)$ and Treatment Effect (TE) based on Equations 4, 5.


(4) $$\begin{array}{l} \mu \left(t\right)=\frac{1}{N}\overset{n}{\underset{1}{\sum }}\{\hat{\alpha_{0}}+\hat{\alpha_{1}}t+\hat{\alpha_{2}}t^{2}+\hat{\alpha_{3}}t^{3}+\hat{\alpha_{4}}\hat{r}\left(t,X_{i}\right)\\ \;+\hat{\alpha_{5}}\hat{r}{\left({t,X}_{i}\right)}^{2}+\hat{\alpha_{6}}\hat{r}{\left(t,X_{i}\right)}^{3}+\hat{\alpha_{7}}t\cdot \hat{r}\left(t,X_{i}\right)\} \end{array}$$

(5) $$TE\left(t\right)=\mu \left(t\right)-\mu \left(0\right),t=0.001,0.002,0.003\ldots 0.99,1$$

Where, N is the sample size. When using the computer to estimate the function μ(t), it is necessary to set the specific value in the interval of [0, 1]. The step size set in this study is 0.01, that is, *t* = 0.001, 0.002, 0.003… 0.99, 1 A total of 101 processing intensity values (50).

## 4 Empirical results

### 4.1 The non-linear impact of commuting time on self-rated health

Table 2 displays the principal estimated results of OLS regression model (Equation 1). Without control variables, the relationships between commuting time and self-rated health are estimated in column (1). In accordance with the previous theoretical analysis, longer commuting time reduces self-rated health. This study controls sociodemographic characteristics of participants for commuting time in column (2) to reduce the non-randomness of commuting time. *Commuting time* remains significant and un favorable, indicating that same conclusion could be drawn. In addition, after the quadratic term for commuting time was introduced in column (3), the coefficient for commuting time turned positive, while the coefficient for the quadratic term was significantly negative. This finding suggests a U-shaped relationship between commuting time and respondents’ self-rated health.

**Table 2:** Effects of commuting time on self-rated health.

Variables	Self-rated health		
	(1)	(2)	(3)
Commuting time	−0.007*** (0.002)	−0.005** (0.002)	0.020** (0.009)
Commuting time 2			−0.000*** (0.000)
Control variable	NO	Yes	Yes
Constant	3.514*** (0.085)	3.109*** (0.356)	2.752*** (0.376)
Observations	1755	1755	1755
R-squared	0.113	0.248	0.258

Standard errors in parentheses.

***p < 0.01, **p < 0.05, *p < 0.1.

The initial improvement observed can be attributed to the potential benefits of short commutes, which may not significantly impact health negatively and might even provide additional physical activity. However, as commuting duration continues to increase, these benefits diminish and the negative impacts become more pronounced, demonstrating a nonlinear relationship. This finding preliminarily validates Hypothesis 1.

**Figure 2:**
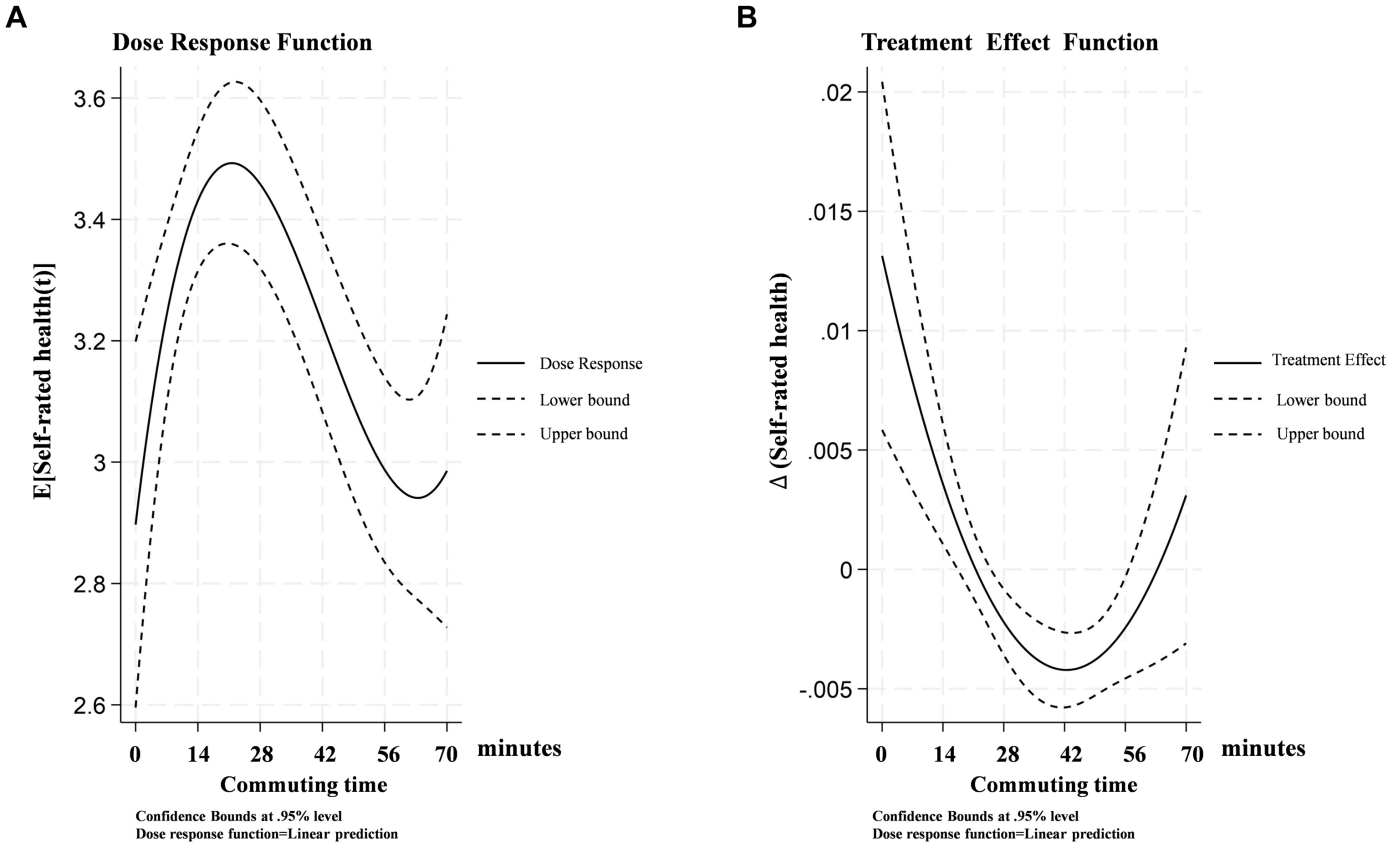
Dose–response function of commuting time on self-rated health.

GPSM was introduced to further estimate the optimal time thresholds and their causal effects on self-rated health across various commuting durations. Figure 2A presents the average “dose–response” function, while Figure 2B reports the effects of varying commuting durations on self-rated health (i.e., treatment effects). It is noteworthy that when the average commuting time exceeds 63 min, the upper and lower bounds of the 95% confidence interval of the dose–response function expand, challenging the statistical significance of commuting duration’s impact on self-rated health. This is primarily due to the small sample size of respondents commuting over an hour constituting only 13% of the total sample. Nevertheless, this does not undermine the conclusion of an inverted “U-shaped” relationship between commuting duration and self-rated health.

In Figure 2A, there is a distinct “N-shaped” relationship between commuting duration and self-rated health, where commuting times within the (0, 21] minute interval have a positive effect on self-rated health, reaching the “location of the optimal dose” (51) [=exp(3.50)] at 21 min. This duration is close to the “happiness commute” time, suggesting that shorter commutes have less impact on individual health, or that people may more easily adapt to this level of commuting (63). However, beyond 21 min, the impact on self-rated health gradually becomes negative, especially when commuting time approaches 42 min (as shown in Figure 2B), where the treatment effect reaches its lowest point, indicating the greatest negative impact. Subsequently, the negative effects gradually diminish, reaching their lowest at 60 min [=exp(2.95)].

This shift confirms that commuting times exceeding a certain threshold may induce significant physical and psychological burdens, including increased fatigue and reduced rest time (52).

Before delving into further exploration of heterogeneity and mechanism analysis, we conducted robustness checks to ensure the accuracy of our research findings. First, this study reassessed core variables by replacing self-rated health with indicators of physical discomfort related to chronic diseases, poor sleep quality, general fatigue, and lack of concentration. Higher values on this scale indicated greater physical discomfort. The regression results, summarized in Table 3, show positive coefficients, suggesting that longer commuting times significantly increase physical discomfort, confirming the robustness of the findings.

**Table 3:** Effects of commuting on physical discomfort.

	Physical discomfort
Commuting time	0.004*
	(0.003)
Control variable	Yes
Constant	1.177***
	(0.400)
*N*	833
R-squared	0.030

***p < 0.01, *p < 0.1.

Second, due to the potential endogeneity and reverse causality between commuting time and self-rated health, the 2SLS estimation method with IV was employed to estimate the causal relationship. Commuting distance was selected as the IV, as it is considered a significant determinant of commuting time while having minimal correlation with self-rated health. The 2SLS results from Table 4 indicate a strong correlation between commuting distance and the endogenous variables, confirmed by a first-stage regression *p*-value under 0.05, validating the instrumental variable’s effectiveness. Subsequent analyses involved regression of the dependent variable on first-stage fitted values, passing under-identification and weak instrument tests with a *p*-value of 59.32, surpassing the Stock-Yogo 10% critical value of 16.38, affirming the instrumental variable’s adequacy.

**Table 4:** 2SLS estimation results of the impact of commuting time on self-rated health.

	IV. Commuting distance
Commuting time	0.063*** (0.000)
*N*	833
R^2	0.101
Anderson canon. corr. LM	56.46
	(0.000)
Cragg-Donald Wald F	59.32
	[16.38]

Values in square brackets are the critical values at the 10% level for the Stock-Yogo weak instrument variable test. The null hypothesis for the Anderson canonical correlation LM test is insufficient instrument variable identification; for the Cragg-Donald Wald F test, the null hypothesis is weak identification of the instrument variable. ***p < 0.01.

### 4.2 Heterogeneity analysis and moderating mechanism

To provide a comprehensive analysis, we compared self-rated health outcomes with variables such as age, sex, and income. Using multicollinearity diagnostics, specifically the Variance Inflation Factor (VIF), we assessed the independence of these variables. The results indicated that all variables had VIF values below 10, suggesting no severe multicollinearity and confirming these variables as independent. We further explored the heterogeneous effects of commuting time on self-rated health across different demographic groups, focusing on gender, age, and income.

(1) Heterogeneous effects with respect to gender

**Figure 3:**
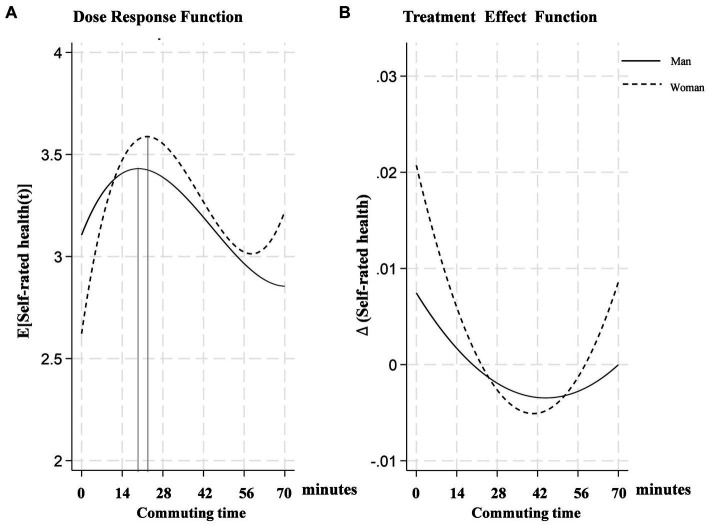
Gender-heterogeneous dose–response function of commuting time on self-rated health.

This paper investigates the gender-specific effects of commuting time on self-rated health, as illustrated in Figure 3. Figure 3A demonstrates an inverted “U” shape trajectory, indicating an initial rise followed by a decline in self-rated health with increased commuting duration. The decline point for men occurs slightly earlier at 20 min compared to 22 min for women. Figure 3B illustrates that women are noticeably more sensitive to commuting time, exhibiting greater fluctuations in self-rated health. Their negative impacts begin to gradually diminish around 41 min, whereas for men, this reduction starts at 46 min.

The observed differences in sensitivity to commuting times between genders can be attributed to various social, psychological, and biological factors. Women’s heightened sensitivity to commuting times may stem from the stress of balancing work with disproportionate domestic responsibilities (25), exacerbating the effects of commuting and leading to greater fluctuations in their self-rated health. Moreover, the distinct points at which significant health declines occur for each gender may reflect differences in stress and fatigue management associated with long commutes. These gender-specific variations underscore the need for targeted interventions to address the unique challenges faced by men and women in relation to commuting (1).

(2) Heterogeneous effects with respect to age

**Figure 4:**
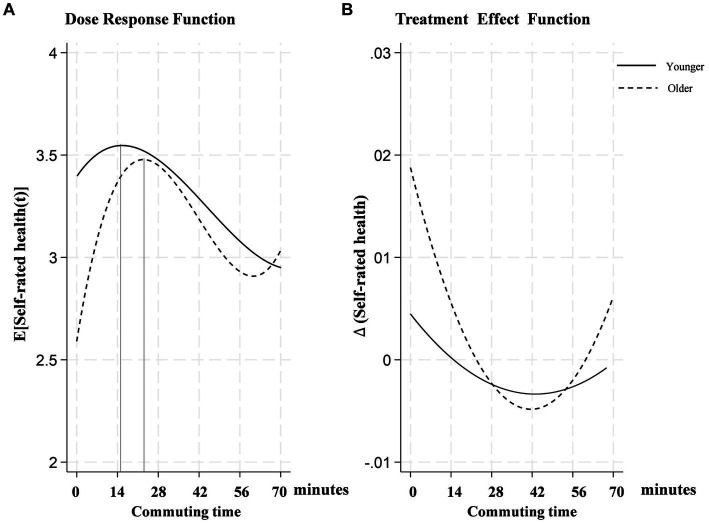
Age-heterogeneous dose–response function of commuting time on self-rated health.

Studies in fields such as physiology and gerontology have shown that different age groups perceive physical exertion and health differently (53). Commuting is a daily physical exertion event, and examining how different age groups respond to this exertion could provide meaningful insights into health impacts. This study categorizes participants into two age groups: those younger and those older than 45 years old. Figure 4A illustrates the dose–response function of commuting time on self-rated health across age groups, with both young adults and older adults showing an inverted “U-shaped” trend. However, the young adult group starts with a higher initial health rating and reaches the peak earlier, suggesting that young people initially cope better with increased commuting times but also experience the adverse effects sooner.

In the treatment effect function depicted in Figure 4B, the curve for the middle-aged and older adults declines earlier and more steeply, indicating a greater sensitivity to increased commuting times. This might suggest that for middle-aged and older adults, the impact of prolonged commuting on health is more immediate and severe. The reduced physiological resilience and lower capacity to recover from physical exertion among older adults make them particularly vulnerable to the stresses associated with long commutes (54).

(3) Heterogeneous effects with respect to income

**Figure 5:**
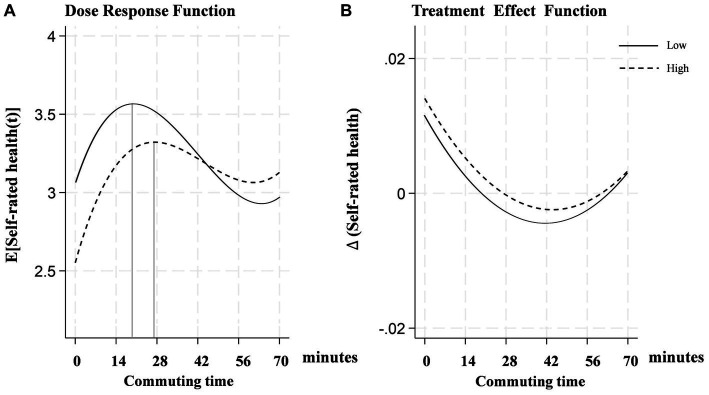
Income-heterogeneous dose–response function of commuting time on self-rated health.

Income levels exert a significant influence on individuals’ perception of the impact of commuting time on their health. In this study, income levels are categorized as high or low, with high income defined as exceeding the median income. Analysis of the dose–response function in Figure 5A reveals that the turning point for the middle and low-income groups (19 min) precedes that of the high-income group (27 min), indicating that economically disadvantaged individuals experience the negative effects of longer commuting times earlier. In the treatment effect function shown in Figure 5B, the decline in health impact is more significant in the low—and middle-income group than in the high-income group. This indicates that higher-income groups may have better resources to alleviate commuting stress, such as more comfortable modes of transportation or more flexible work arrangements (10).

The disparities in the impacts of commuting on health across income levels can be largely attributed to differences in commuting conditions, job flexibility, and access to healthcare. Higher-income individuals often enjoy more comfortable commuting options and have jobs that allow for telecommuting or flexible hours, reducing commute-related stress (55). They also tend to live in areas closer to work with better infrastructure, further alleviating the daily burden of commuting. Additionally, their greater access to healthcare helps manage any adverse effects of commuting, contributing to better overall health outcomes compared to lower-income counterparts (56).

### 4.3 Moderating mechanism analysis

(1) Moderating role of active commuting and physical activity

This study further examines their moderating mechanisms on the commuting-health relationship. Results from Table 5 column (3) reaffirm the beneficial effects of both active commuting and physical activity. However, while physical activity continues to positively moderate the relationship between commuting time and health, its direct impact has become non-significant. This suggests that the frequent dispersal of physical activity throughout the day might dilute its immediate, noticeable benefits on health metrics, reducing its statistical significance in models analyzing instant health outcomes. Intriguingly, the moderating effect of active commuting is negative, indicating that despite the general health benefits of active commuting methods such as walking or cycling, their integration into longer commuting durations may introduce additional physical strain or stress. This negative effect is particularly pronounced when commuting infrastructure is inadequate (e.g., poor cycling lanes, severe traffic congestion) or when commuting exceeds certain thresholds, potentially offsetting the health benefits.

(2) Moderating role of perceived built environment at residential and workplace settings

**Table 5:** Results of moderating effects.

	(1)	(2)	(3)	(4)
Variables	Self-rated health			
Commuting time	−0.421***	−0.441*** (0.145)	−0.456*** (0.144)	−0.472*** (0.144)
	(0.138)			
Active commuting	0.417***	0.376*** (0.037)		
	(0.037)			
Commuting time* Mode of commuting		−0.533*** (0.145)		
Physical activity	0.010* (0.010)	0.015 (0.009)		
Commuting time* Physical activity		0.061* (0.035)		
Workplace Green Space			−0.114 (0.069)	−0.020 (0.037)
Commuting time* Workplace Green Space				0.135 (0.131)
Workplace Walkability			0.090 (0.065)	0.023 (0.035)
Commuting time* Workplace Walkability				0.172 (0.120)
Workplace Safety			−0.030 (0.064)	−0.002 (0.044)
Commuting time* Workplace Safety				0.328** (0.145)
Workplace Accessibility			0.047 (0.047)	0.039 (0.033)
Commuting time* Workplace Accessibility				0.148 (0.109)
Residential Green Space			−0.095 (0.077)	−0.032 (0.036)
Commuting time* Residential Green Space				0.312** (0.121)
Residential Walkability			0.261** (0.123)	0.251** (0.123)
Commuting time* Residential Walkability				0.251** (0.123)
Residential Safety			0.023 (0.067)	0.001 (0.039)
Commuting time* Residential Safety				0.351*** (0.134)
Residential Accessibility			0.128*** (0.048)	0.068** (0.033)
Commuting time*Residential Access				0.150 (0.112)
Control variable	Yes	Yes	Yes	Yes
Constant	2.788*** (0.330)	2.814*** (0.347)	2.836*** (0.346)	2.801*** (0.349)
Observations	1,755	1,755	1,755	1,755
R-squared	0.341	0.341	0.345	0.344

Robust standard errors in parentheses.

***p < 0.01, **p < 0.05, *p < 0.1.

This study delves deeper into the moderating mechanisms of perceived built environments on the commuting-health relationship. According to the results presented in Table 5 column (3), perceived environments at workplaces do not significantly influence health, possibly due to the limited outdoor interaction as most work-related activities occur indoors. However, residential environments characterized by perceived walkability and accessibility show a positive effect on health, underscoring the direct impact of residential environmental quality on daily activity levels and health, especially in facilitating physical activities and convenient daily commutes (57).

Furthermore, Table 5 column (4) reveals that safety perceptions at both workplace and residential settings, along with perceived residential greenness, do not directly affect health outcomes. However, they do positively modulate the commuting-health relationship. This modulation is attributed to the psychological comfort provided by greener and safer living environments, which helps alleviate the stress associated with commuting (58). Notably, perceived residential accessibility, despite its direct health benefits, does not modify the relationship between commuting and health. This is because, while residential accessibility enhances the convenience of daily life, its role in alleviating the complex impact of increased commuting times on health may not be significant.

## 5 Conclusion and discussion

This study delves into the nonlinear causal relationship between long-duration commuting and health outcomes, moving beyond the correlational focus prevalent in previous research. This study contributed three original contributions to the body of knowledge. First, this study not only confirms the impact of long-duration commuting on health outcomes but also establishes critical causal time thresholds, delineating when negative effects begin and the point at which they become severely detrimental. Second, the study reveals that the effects of long-duration commuting vary significantly across different genders, ages, and income levels, highlighting the importance of identifying particularly vulnerable groups. Third, the study enhances existing literature by demonstrating how individual behaviors and environmental contexts significantly influence health outcomes associated with commuting.

This study infers the causal relationship between commuting time and health outcomes by employing control variables and designing randomized experiments, effectively mitigating the selection and confounding biases commonly encountered in empirical research. These biases often arise from variations in residents’ commuting distances, socioeconomic factors, and car ownership. This rigorous methodology ensures a clearer identification of causal links. This study demonstrates that while commuting durations up to 21 min can positively influence health, exceeding this threshold progressively harms well-being, especially past 42 and 60 min. This nuanced delineation of critical time points advances the discussion beyond the simplistic 60-min benchmark prevalent in previous studies (59), fostering a more comprehensive understanding of the causal dynamics that may spur further research and debate. Furthermore, the study confirms that commuting, when perceived as beneficial physical activity within defined limits, significantly contributes to daily health maintenance. This insight is essential for urban planners and public health officials as they devise interventions aimed at maximizing the health benefits of active commuting while avoiding the detrimental effects that emerge beyond established thresholds.

Heterogeneity analysis yields profound insights, showing that compared to men and younger adults, women and older adults demonstrate a significantly higher sensitivity to long-duration commuting. Interestingly, their threshold for experiencing negative effects is delayed. This discrepancy is largely due to their greater initial resilience, adaptive coping mechanisms, and robust social and commuting habits, which collectively cushion the early impacts of extended commuting times (5). Similarly, while low-income groups may not show significant initial sensitivity to long-distance commuting, they encounter negative health impacts much earlier than middle and high-income groups. This early onset of negative effects among low-income groups stems from their limited resources to manage commuting stress and pre-existing socioeconomic health vulnerabilities, highlighting the urgent need for targeted interventions to effectively support these groups (60). These findings emphasize the complex interplay between socioeconomic status, age, and gender in commuting-related health impacts, underscoring the necessity for nuanced policy interventions tailored to diverse community needs.

This study explored how behavioral preference, and perceived built environments act as moderators in the health-commuting relationship, confirming their general benefits but revealing complex dynamics. Regular physical activities and active commuting methods such as walking and cycling generally promote health (35). However, while regular physical activity can positively influence the dynamics between commuting and health, its direct impact on specific health metrics often becomes statistically insignificant when analyzed in isolation. Furthermore, integrating active commuting into longer commute durations can introduce additional strain. This complexity suggests that the benefits of active commuting can be offset by the increased physical demands and stress associated with prolonged commuting times, highlighting the need for balanced approaches in promoting active commuting as a health-enhancing behavior (1). Additionally, while residential settings with better walkability and accessibility enhance health, workplace environmental factors show negligible effects. The modulation by safer and greener residential areas highlights the importance of environmental quality in mitigating commuting stress (61), though increased accessibility alone does not significantly alter the health impacts of longer commutes, underscoring the nuanced role of urban planning in enhancing public health through careful consideration of commuting practices and environmental settings.

This study’s insights into commuting and health are limited by its reliance on self-reported data, which may introduce biases, and its focus on self-rated health as an indicator, rather than a comprehensive measure of individual health. The specific contexts examined may also affect the generalizability of the results. Future research could benefit from using objective data and examining broader geographic settings to enhance reliability and applicability. Additionally, the use of a scoring system to quantify the relative physical activity levels associated with different commuting modes presents limitations. While the scoring system aims to reflect general trends where more active commuting modes contribute to higher physical activity levels, the actual intensity and duration of physical activity can vary significantly among individuals and contexts. Therefore, the scores should be viewed as relative measures rather than exact multipliers of physical activity levels. Future research could benefit from using objective data and examining broader geographic settings to enhance reliability and applicability. Further exploration of long-term impacts and environmental factors will be crucial for developing targeted urban planning and public health policies.

## Data Availability

The datasets presented in this article are not readily available because the data that support the findings of this study are not publicly available due to privacy and ethical restrictions. Requests to access the datasets should be directed to Ning Qiu, qiuning22@sdjzu.edu.cn.
